# The Differences Between Motor Attempt and Motor Imagery in Brain-Computer Interface Accuracy and Event-Related Desynchronization of Patients With Hemiplegia

**DOI:** 10.3389/fnbot.2021.706630

**Published:** 2021-11-05

**Authors:** Shugeng Chen, Xiaokang Shu, Hewei Wang, Li Ding, Jianghong Fu, Jie Jia

**Affiliations:** ^1^Department of Rehabilitation Medicine, Huashan Hospital, Fudan University, Shanghai, China; ^2^School of Mechanical Engineering, Shanghai Jiao Tong University, Shanghai, China; ^3^National Clinical Research Center for Aging and Medicine, Huashan Hospital, Fudan University, Shanghai, China; ^4^National Center for Neurological Disorders, Shanghai, China

**Keywords:** BCI accuracies, event-related desynchronization, motor attempt, motor imagery, brain-computer interface

## Abstract

**Background:** Motor attempt and motor imagery (MI) are two common motor tasks used in brain-computer interface (BCI). They are widely researched for motor rehabilitation in patients with hemiplegia. The differences between the motor attempt (MA) and MI tasks of patients with hemiplegia can be used to promote BCI application. This study aimed to explore the accuracy of BCI and event-related desynchronization (ERD) between the two tasks.

**Materials and Methods:** We recruited 13 patients with stroke and 3 patients with traumatic brain injury, to perform MA and MI tasks in a self-control design. The BCI accuracies from the bilateral, ipsilesional, and contralesional hemispheres were analyzed and compared between different tasks. The cortical activation patterns were evaluated with ERD and laterality index (LI).

**Results:** The study showed that the BCI accuracies of MA were significantly (*p* < 0.05) higher than MI in the bilateral, ipsilesional, and contralesional hemispheres in the alpha-beta (8–30 Hz) frequency bands. There was no significant difference in ERD and LI between the MA and MI tasks in the 8–30 Hz frequency bands. However, in the MA task, there was a negative correlation between the ERD values in the channel CP1 and ipsilesional hemispheric BCI accuracies (*r* = −0.552, *p* = 0.041, *n* = 14) and a negative correlation between the ERD values in channel CP2 and bilateral hemispheric BCI accuracies (*r* = −0.543, *p* = 0.045, *n* = 14). While in the MI task, there were negative correlations between the ERD values in channel C4 and bilateral hemispheric BCI accuracies (*r* = −0.582, *p* = 0.029, *n* = 14) as well as the contralesional hemispheric BCI accuracies (*r* = −0.657, *p* = 0.011, *n* = 14). As for motor dysfunction, there was a significant positive correlation between the ipsilesional BCI accuracies and FMA scores of the hand part in 8–13 Hz (*r* = 0.565, *p* = 0.035, *n* = 14) in the MA task and a significant positive correlation between the ipsilesional BCI accuracies and FMA scores of the hand part in 13–30 Hz (*r* = 0.558, *p* = 0.038, *n* = 14) in the MI task.

**Conclusion:** The MA task may achieve better BCI accuracy but have similar cortical activations with the MI task. Cortical activation (ERD) may influence the BCI accuracy, which should be carefully considered in the BCI motor rehabilitation of patients with hemiplegia.

## Introduction

Motor attempt and motor imagery (MI) are two common experimental paradigms in the non-invasive electroencephalogram (EEG)-based brain-computer interface (BCI) system design. Motor imagery is a cognitive rehearsal of physical movements that is defined as the internal reactivation of any first-person motor performance without an overt motor output (Jeannerod, 1995, 2001). There is extensive use of MI for athletes. Action observation combined with MI has been shown to engage the motor system in sports (Di Rienzo et al., 2019). Video observation and MI have been used to improve jumping performance in national rhythmic gymnastics athletes (Battaglia et al., 2014). Motor attempt is defined as attempting to move a paralyzed hand with little or no covert movement, specifically for patients with motor disability (Antelis et al., 2017). A meta-analysis by Bai et al. (2020) suggested that using movement attempts as the trigger task in BCI training appeared to be more effective than using MI. A study (Hotz-Boendermaker et al., 2008) of neural activity using functional MRI (fMRI) in paraplegics showed that during the attempt to move, the primary motor cortex is slightly less engaged than during the imagination of movement, however, the regions of the parietal lobe and cerebellum, well known to be involved in sensorimotor integration, are more activated during the attempt to move. For patients paralyzed in the upper limbs after a stroke, attempted movement is more easily detected in EEG than motor imagination (Muralidharan et al., 2011). A study by Blokland et al. (2015) showed the differences between attempted movement and actual movement using a neuromuscular blocker.

Both motor attempt (MA) and MI tasks can induce cortical activations, which can be applied in BCI decoding. Recent research into MA- and MI-BCI has so far yielded positive results. A randomized controlled trial (RCT) reported that MA-BCI could improve hand function in chronic stroke patients after 4 weeks of training (Ramos-Murguialday et al., 2013). Rathee et al. (2019) tried MA-related EEG-driven hand-exoskeleton on post-stroke patients and found improvement in their Action Research Arm Test (ARAT). Pichiorri et al. (2015) found that 1 month of MI-BCI intervention achieved greater power spectra in the alpha and beta bands in the ipsilesional hemisphere and improved motor function in subacute stroke patients with severe motor deficits. Although MA- and MI-BCI have been both widely but, respectively, researched (Bundy et al., 2017), the overall analysis and research on the BCI accuracy of MA tasks have not been done in patients with hemiplegia, especially compared further with MI tasks. Mizuno et al. (2018) proposed a protocol to compare MA-BCI and MI-controlled treatment to explore the efficacy of MA-BCI in stroke patients but no results have been reported yet. Blokland et al. (2014) used EEG and functional near-infrared spectroscopy (fNIRS) to test the feasibility of using MA instead of MI as a task for brain switch control. Which paradigm to choose from MA and MI tasks in BCI testing and training is still uncertain and is an important question to answer.

Brain-computer interface accuracy is an important parameter in BCI-based intervention. Higher BCI accuracies have been correlated with larger excitability in healthy people (Niazi et al., 2012) and better motor recovery in patients with hemiplegia (Biasiucci et al., 2018). Patients with hemiplegia usually presented different cortical excitability from healthy people (Wong et al., 2013; Agius Anastasi et al., 2017; Li et al., 2019), and their cortical activation patterns changed a lot due to cerebral injury (Shu et al., 2019). As a result, the choices of EEG channels have a great influence on BCI decoding effects. Research on BCI accuracies and control varied among the bilateral, ipsilesional, and contralesional hemispheres in patients with hemiplegia. Lopez-Larraz et al. (2017) analyzed EEG from the whole-brain channels while Ramos-Murguialday et al. and Ono et al. collected EEG signals directly from the ipsilesional hemisphere. Their subjects were asked to perform an MA task with their paralyzed hand (Ramos-Murguialday et al., 2013; Ono et al., 2015). Interestingly, Antelis et al. (2017) and Bundy et al. (2017) both achieved reasonable BCI accuracies by decoding EEG signals from the contralesional hemisphere. The BCI accuracies were different between MA and MI tasks in spinal cord injury (SCI) patients. Lopez-Larraz et al. (2012) reported a higher accuracy of MA than MI in SCI patients. Blokland et al. (2014) reported a significantly higher average accuracy for MA than MI in patients with tetraplegia. Although several studies explored the differences in BCI accuracy in SCI patients, it is still unclear how could it be different concerning MA and MI tasks in patients with hemiplegia.

Additionally, event-related de/synchronization (ERD/ERS) are common indexes extracted from EEG during MA and MI tasks (Pfurtscheller et al., 1999; Müller-Putz et al., 2007). A higher magnitude of ERD activity is related to larger cortical activation during motor tasks (Pfurtscheller et al., 1999; Takemi et al., 2013; Kaiser et al., 2014). It is considered that MI is close to attempting movement by the fact that it is linked to kinesthetic motor imagery (KMI) and kinesthetic feeling (Nikulin et al., 2008). Moreover, the ERD following the KMI after learning is very similar to those generated during motor execution or MA (Rimbert et al., 2019a). It was further reported that BCI accuracy was highly associated with mu-band ERD (Kaplan et al., 2016). Cortical activations vary between different motor tasks. Kraeutner et al. (2014) reported that the strength of ERD was significantly greater in motor execution than in MI in non-disabled participants. Higher motor impairment was reported to be related to stronger ERD in the unaffected hemisphere in MI tasks while it was related to the higher hemispheric asymmetry of ERS in motor execution tasks in stroke patients (Kaiser et al., 2012). However, the MI-induced cortical activity change was significantly augmented and even exceeding that of motor execution tasks in controlling a computer cursor (Miller et al., 2010). In addition, primary motor cortices have a symmetrical organization between the right and left hemispheres, particularly in hand motor control (Cicinelli et al., 1997; Tecchio et al., 1997; Del Gratta et al., 2000). In fMRI-based neuroscience research, the laterality index (LI) (Caria et al., 2011) was used to measure the inter-hemispheric balance in cortical activations (Pivik et al., 1993). Ramos-Murguialday et al. (2013) used LI in an MA-BCI study with fMRI to show the different activations between hemispheres. Johnson et al. (2018) combined repetitive transcranial magnetic stimulation (rTMS) and MI-BCI in stroke and found significant alterations in the interhemispheric inhibition and increased relative ipsilesional cortical activation. However, the inter-hemispheric balance has not yet been compared directly between MA and MI tasks. The differences in ERD/ERS, as well as LI between MA and MI tasks in patients with hemiplegia, need to be further explored. The findings in the current study may provide references on how to choose different BCI experimental paradigms (MA or MI tasks) in BCI training.

Given the lack of studies investigating BCI accuracy and the EEG features between MA and MI tasks in patients with hemiplegia, we aim to explore the cortical difference between MA and MI tasks. We will calculate the BCI accuracy across the hemispheres and ERD values. We hypothesize that there is both difference and relationship in BCI accuracy and ERD of patients with hemiplegia.

## Materials and Methods

### Subject Recruitment

Sixteen patients were recruited from the Department of Rehabilitation Medicine of Huashan Hospital. The EEG data of two patients were contaminated with large artifacts and were discarded. The remaining 14 of the 16 patients (age: 45.7 ± 15.1 years) were enrolled in the further analysis. All the patients met the following inclusion criteria: (1) first-time unilateral stroke who are >2 weeks post-stroke and confirmed by scan or diagnosed with a unilateral traumatic brain injury and in the rehabilitation stage; (2) aged from 25 to 70 years; (3) right-handed; (4) mini-mental state examination (MMSE) ≥ 25; and (5) was able to sit independently in a chair for at least 1 h. The exclusion criteria included: (1) had unilateral neglect or vision problem; (2) receiving non-invasive brain stimulation during the study; (3) allergic to electrode gel; and (4) had previous experience with or knowledge of MA and MI tasks. Participant demographics and clinical characteristics are shown in Table 1. This study was approved by the Ethical Committee of Huashan Hospital. Informed consent was signed according to the Declaration of Helsinki.

**TABLE 1:** Demographics and clinical characteristics of the patients.

**Patients**	**Age (yrs)**	**AH**	**TI**	**SI**	**Injury location**	**TSI (m)**	**MMSE**	**FMA-UL (max = 66)**	**FMA-hand (max = 24)**
P1	66–70	R	I	S	Basal ganglia	84	29	12	1
P2	61–65	L	I	C+S	Basal ganglia, corona radiata, frontal cortex	4	30	10	0
P3	25–30	R	TBI	C	Parietal cortex	132	30	53	15
P4	31–35	L	I	S	Basal ganglia	16	30	24	2
P5	56–60	R	H	S	Basal ganglia	11	28	4	0
P6	41–45	R	H	S	Basal ganglia	1	30	50	15
P7	61–65	L	I	C+S	Basal ganglia, thalamus, paracele, frontoparietal cortex	8	30	12	1
P8	46–50	R	H	S	Basal ganglia	12	25	18	1
P9	46–50	R	I	C	Frontal, parietal, temporal cortex	1	30	37	13
P10	25–30	L	TBI	C	Subdural	21	30	24	1
P11	31–35	R	TBI	C	Parietal cortex	180	30	34	2
P12	36–40	R	I	S	Insular lobe	5	30	29	1
P13	61–65	R	I	S	Brainstem	3	30	13	1
P14	25–30	R	H	S	Frontoparietal cortex	28	30	49	12

*AH, affected hand; TI, type of injury; SI, site of injury; TSI, time since injury; R, right; L, left; S, Subcortical; C+S, Cortical and subcortical; C, cortical; I, ischemia; H, hemorrhage; TBI, traumatic brain injury; yrs, years; m, months.*

### Experimental Design and Electroencephalogram Data Collection

**FIGURE 1:**
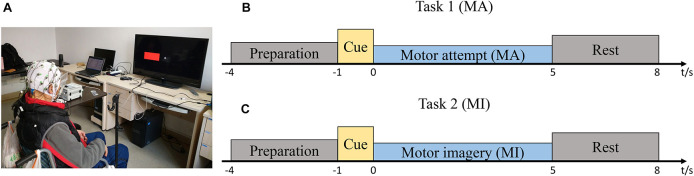
Study setup and experimental protocol. **(A)** The patient was seated in a chair in front of a screen. **(B)** Timeline of a single trial during the motor attempt (MA) task. **(C)** Timeline of a single trial during the motor imagery (MI) task.

The patients were asked to sit on a chair/wheelchair in front of a screen in a comfortable posture (Figure 1A). An EEG cap (actiCAP, Brain Products, Germany) consisting of 32 channels of Ag/AgCl electrodes was used for EEG recording. The electrodes were distributed according to the 10–20 international system (Klem et al., 1999). The reference channel was placed on the right mastoid process and the ground channel was placed on the forehead. The impedance was kept below 5 kΩ. The signals were amplified with BrainAmp (Brain Products, Gilching, Germany) and recorded at a sampling rate of 200 Hz. The raw EEG signals were filtered with a bandpass filter of [1, 100] Hz.

The patients were asked to perform two sessions of MA or MI tasks of wrist extension. One session included 15 right-hand trials and 15-left hand trials and the trial types (right or left) appeared randomly. In one session, there was only one type of task. The MA and MI tasks were done randomly on different days for the same patient to avoid possible temporal effects from the order of the two tasks. In the MI task, the patients imagined extending the wrists without overt motor output; in the MA task, the patients tried to perform the wrist extension. For both motor tasks, there was a pre-training session before the formal testing. In the pre-training session, the patients were required to perform the MA or MI tasks with both affected and unaffected hands simultaneously. During the MA tasks, visible movements could be observed from the unaffected hand. During the MI tasks, no covert movements were observed from neither affected nor unaffected hands. When they performed the MA tasks with the affected hands, not all wrist extension movements were visible, but they were told to try their best.

Figures 1B,C show the timelines of a single trial during MA/MI tasks. Prior to EEG recording, all the patients practiced the MA or MI tasks and became familiar with the cues on the screen. At the beginning of each trial, a white “+” appeared at the center of the screen to remind the patients to prepare for the task. After 3 s, a red rectangle appeared to inform the patients to perform the motor tasks according to the left or right cue. The red rectangle then disappeared after 1 s and the patients began to perform the MA or MI tasks for 5 s until the white “+” disappeared. During the 5 s, they attempted to perform sustained wrist extension (Cassim et al., 2000). After that, there was a resting period to reduce the chance of the adaptation of the patients. The resting period was randomized to last between 2 and 3 s. The recording session was kept relatively short to minimize discomfort and to ensure the patients were focused on the tasks. The patients were required to look at a stationary fixation point at the center of the screen to minimize eye movement artifacts. They were also instructed to avoid excessive eye blinking, swallowing, or any irrelevant movement.

### Data Pre-processing

The left hemisphere was covered with FP1, FZ, F3, F7, FT9, FC5, FC1, C3, T7, TP9, CP5, CP1, PZ, P3, and P7 (15 channels) while the right hemisphere was covered with O2, P4, P8, TP10, CP6, CP2, CZ, C4, T8, FT10, FC6, FC2, F4, F8, and FP2 (15 channels). For the tasks with affected hands (vs. rest), the BCI accuracies of the bilateral hemispheres were calculated with 31 channels except for the reference channel (the 32 channel). The BCI accuracies of the right or left hemisphere were calculated with 15 channels, respectively. The average ERD/ERS in the bilateral, ipsilesional, and contralesional hemispheres were also calculated based on the same number of channels.

The preprocessed EEG data consisted of high-pass filtering at 1 Hz and low-pass filtering at 30 Hz. Then, the datasets were subjected to an independent component analysis (ICA) decomposition by using EEGLAB (Delorme and Makeig, 2004). The ICA components representing eyeblink, head movement, and power line interference were removed from the data. Manual checking was performed in the EEG data of all 31 channels and all trials. No bad channel and bad trial rejection were performed in the data.

### Brain-Computer Interface Accuracy Calculation

The offline BCI accuracies were evaluated by the single-trial decoding accuracy between the task and idle states. In every single trial, the task state was defined at [1, 4] s, and the idle state was defined at [−4, −1] s. The EEG features were extracted with the common spatial pattern (CSP) algorithm (Benjamin et al., 2008). The log-variance of the first and last three components produced by the CSP filters were selected as feature vectors. They were subsequently classified using linear discriminative analysis (LDA). The two classes (MA vs. rest and MI vs. rest) of the affected hand were classified in the offline analysis. The EEG features were extracted from the alpha-beta frequency bands (8–30 Hz).

In the offline analysis, 10-fold cross-validation was conducted with the dataset for each experimental condition. All 31 channels of the EEG signals were used for pattern classification. The 30 trials of the task states and 30 trials of the idle states of the affected hand were randomly divided into 10 sets. Each set was tested with the classifier which was calibrated using the other nine sets. This analysis was repeated 10 times, generating 100 decoding accuracies. The EEG features were also extracted from the alpha-beta frequency bands using the CSP filters. The BCI accuracies of all 14 patients were evaluated with the average classification accuracy and SD. The detailed calculation formulas of the BCI accuracy can be referred to in the published paper (Yao et al., 2013).

### Event-Related Desynchronization/Event-Related Synchronization Values Analysis

For each channel, we computed the power spectrum at the alpha-beta frequency bands (8–30 Hz) to identify the ERD/ERS on the motor tasks of the affected hand. The time-frequency distributions (TFDs) of the EEG trials were estimated using a windowed Fourier transform (WFT) (Peng et al., 2019) with a fixed 200-ms Hanning window. The WFT yielded, for each trial, a complex time-frequency estimate F(t,f) at each time-frequency point (t,f), extending from -3,000 to 5,000 ms (in steps of 5 ms) in the time domain, and from 1 to 30 Hz (in steps of 1 Hz) in the frequency domain. The power spectrum (P), *P*(t,f) = | F(t,f)| ^2^, was obtained. The percentage of the relative power decrease was calculated to obtain the ERD/ERS with respect to a resting-state baseline ([−3, −1] s). The interest time was set at [1, 4] s, during which the patient was performing the MA or MI tasks. For that, the ERD/ERS in the alpha-beta (8–30 Hz) frequency bands were averaged in the time interval [1, 4] s. The formula of the ERD/ERS (Pfurtscheller et al., 1999) is:

$$\mathrm{ERD}/\mathrm{ERS}=\frac{P_{i\,n\,t\,e\,r\,e\,s\,t}-P_{b\,a\,s\,e\,l\,i\,n\,e}}{P_{b\,a\,s\,e\,l\,i\,n\,e}}*100\%$$

By using this definition, ERD was expressed as a negative value and stronger ERD is related to higher cortical activations during the motor tasks (MA or MI) (Pfurtscheller et al., 1999). Laplace transformation was applied when calculating the correlations between the ERD and BCI accuracies. The cortical positions of the patients with injury in the right hemispheres were flipped for calculating the ERD values, simulating that all the patients have an injury in the left hemispheres. The topographies were drawn with an interest time of 1 to 4 s, with respect to a resting-state baseline ([−3, −1] s). The time-frequency maps were drawn with the above-mentioned calculation, representing the signal magnitude as a joint function of time and frequency at each time-frequency point.

The LI, approaching a value of 1 or −1 when the brain activity was either purely ipsilesional or contralesional (Caria et al., 2011), was calculated from the ERD values in both the ipsilesional and contralesional hemispheres during the interest time when the patient was performing the motor tasks. The formula of LI is:

$$\text{LI}=\frac{E\,R\,D_{i\,p\,s\,i\,l\,e\,s\,i\,o\,n\,a\,l}-E\,R\,D_{c\,o\,n\,t\,r\,a\,l\,e\,s\,i\,o\,n\,a\,l}}{\left|E\,R\,D_{i\,p\,s\,i\,l\,e\,s\,i\,o\,n\,a\,l}\right|+\left|E\,R\,D_{c\,o\,n\,t\,r\,a\,l\,e\,s\,i\,o\,n\,a\,l}\right|}$$

### Data Analysis

The statistical analysis was performed with SPSS version 19.0 (SPSS Inc., Chicago, IL, United States) and the figures were drawn with GraphPad Prism 7 Software (GraphPad Software, Inc., San Diego, CA, United States). Two-way repeated-measures ANOVA, taking both task (two levels: MA and MI tasks) and hemisphere (three levels: bilateral, ipsilesional, and contralesional hemispheres) as the within-subject factors, were performed on the BCI accuracies and ERD values. A paired *t*-test was applied as a *post hoc* analysis and was used to compare the LI values between the MA and MI tasks. Spearman correlation was used between the BCI accuracies and ERD/ERS, between the BCI accuracies and FMA scores, and the ERD/ERS between the tasks. The statistical significance was set at *p* < 0.05. Bonferroni correction was applied in multiple comparisons.

## Results

### Comparison of Brain-Computer Interface Accuracies Between Motor Attempt and Motor Imagery

**FIGURE 2:**
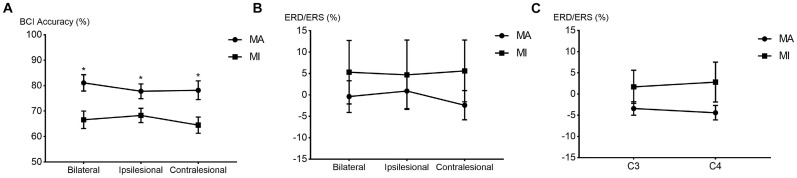
The line chart and scatter plot of brain-computer interface (BCI) accuracies and event-related de/synchronization (ERD/ERS) values in the 8–30 Hz band. **(A)** The average BCI accuracies (mean ± SEM) between the MA and MI tasks in the bilateral, ipsilesional, and contralesional hemispheres. *p < 0.0167 after Bonferroni correction. **(B)** The average ERD/ERS values (mean ± SEM) between the MA and MI tasks in the bilateral, ipsilesional, and contralesional hemispheres. **(C)** The average ERD/ERS values (mean ± SEM) between the MA and MI tasks in the C3/C4 channels. SEM, standard error of mean.

The comparison of BCI accuracies of 14 patients in the 8–30 Hz band is shown in Figure 2A. The main effect analysis from the two-way repeated-measures ANOVA on the BCI accuracies showed that the tasks had a significant effect on BCI accuracies (*F*_1,13_ = 13.293, *p* = 0.003) while there was no significant effect for the hemispheres on BCI accuracies (*F*_2,26_ = 1.49, *p* = 0.244). There was no significant hemisphere × task interaction (*F*_2,78_ = 2.441, *p* = 0.107). The estimated marginal means showed an average BCI accuracy of 79% (72.3–85.7% in 95% CI) in the MA task and an average BCI accuracy of 66.5% (60.1–72.8% in 95% CI) in the MI task. The BCI accuracy in the MA task was 12.6% (5.1–20% in 95% CI) higher than that in the MI task. Table 2 shows the BCI accuracy and variance of accuracy for each patient in the MA and MI tasks, respectively.

**TABLE 2:** The BCI accuracy and variance of accuracy for each patient in the MA and MI tasks, respectively.

**MA task**		**P1**	**P2**	**P3**	**P4**	**P5**	**P6**	**P7**	**P8**	**P9**	**P10**	**P11**	**P12**	**P13**	**P14**
Bilateral H	mean	0.753	0.910	0.885	0.807	0.917	0.891	0.667	0.676	0.651	0.957	0.645	0.930	0.943	0.723
	SD	0.077	0.096	0.058	0.089	0.080	0.065	0.108	0.131	0.087	0.049	0.147	0.067	0.052	0.124
Ipsilesional H	mean	0.709	0.927	0.743	0.793	0.810	0.782	0.693	0.631	0.698	0.943	0.725	0.907	0.910	0.617
	SD	0.111	0.065	0.078	0.067	0.092	0.100	0.129	0.103	0.126	0.058	0.130	0.060	0.086	0.132
Contralesional H	mean	0.820	0.860	0.820	0.758	0.843	0.831	0.597	0.704	0.642	0.957	0.505	0.943	0.957	0.707
	SD	0.076	0.124	0.079	0.096	0.108	0.119	0.142	0.100	0.100	0.042	0.151	0.062	0.049	0.108

**MI task**		**P1**	**P2**	**P3**	**P4**	**P5**	**P6**	**P7**	**P8**	**P9**	**P10**	**P11**	**P12**	**P13**	**P14**

Bilateral H	mean	0.613	0.850	0.530	0.543	0.567	0.743	0.593	0.645	0.640	0.600	0.560	0.773	0.883	0.837
	SD	0.127	0.087	0.122	0.116	0.102	0.115	0.119	0.108	0.110	0.142	0.128	0.117	0.080	0.098
Ipsilesional H	mean	0.600	0.883	0.570	0.627	0.647	0.870	0.647	0.643	0.645	0.573	0.578	0.777	0.783	0.717
	SD	0.105	0.083	0.129	0.154	0.097	0.072	0.126	0.120	0.122	0.121	0.101	0.089	0.108	0.113
Contralesional H	mean	0.597	0.780	0.533	0.610	0.530	0.717	0.560	0.695	0.585	0.617	0.502	0.703	0.810	0.860
	SD	0.159	0.093	0.102	0.124	0.102	0.113	0.119	0.077	0.086	0.113	0.118	0.099	0.128	0.086

*H, hemisphere; SD, standard deviation.*

## Evaluation of Cortical Activations Between Motor Attempt and Motor Imagery

The main effect analysis from the two-way repeated-measures ANOVA on the ERD/ERS of the 14 patients showed that the tasks had no significant effect on ERD/ERS and no significant effect for the hemispheres on ERD/ERS. There was also no significant hemisphere × task interaction. There was no significant difference between the MA and MI tasks in the average ERD/ERS with the bilateral or unilateral hemispheres. The main effect analysis from the two-way repeated-measures ANOVA on the C3/C4 ERD/ERS of the 14 patients showed no significant main effect. There was also no significant hemisphere × task interaction. Figures 2B,C show the line chart and scatter plot of the ERD/ERS values between the MA and MI tasks in the 8–30 Hz band.

**FIGURE 3:**
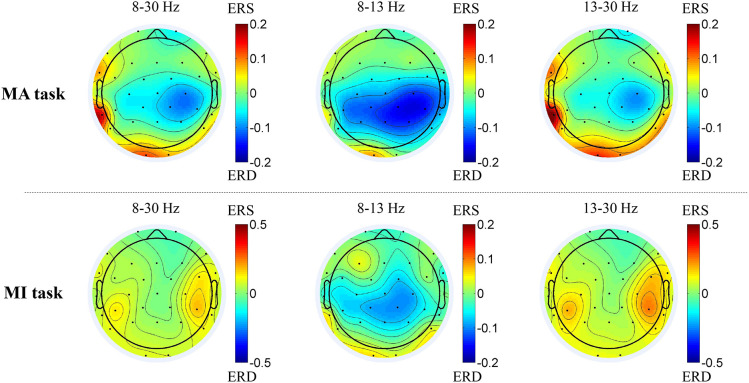
Average topographies of all 14 patients in the 8–30 Hz, 8–13 Hz and 13–30 Hz frequency bands between the MA and MI tasks. The first row shows the average topographies of the 8–13 Hz and 13–30 Hz bands in the MA tasks. The second shows the average topographies of the 8–30 Hz, 8–13 Hz and 13–30 Hz bands in the MI tasks.

Figure 3 shows the average topographies of all 14 patients in the alpha-beta frequency bands between the MA and MI tasks. In the 8–13 Hz band, the patients presented strong activations (ERD, with color blue) in both tasks while the MA task was stronger. In 8–30 and 13–30 Hz bands, both ERD and ERS existed.

**FIGURE 4:**
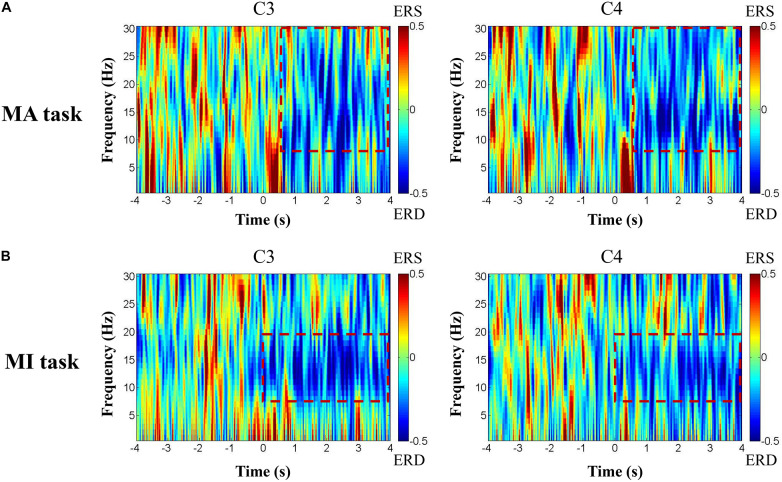
The ERD pattern changes over time of one patient with left hemisphere injury during the motor tasks. The ERD was presented in the red rectangular box in 8–30 Hz frequency bands in channels C3 and C4 of the MA task **(A)** and 8–13 Hz frequency bands in channels C3 and C4 of the MI task **(B)**.

Figure 4 shows the ERD pattern changes over time for one patient with left hemisphere injury during the motor tasks. The ERD was presented in the red rectangular box in the 8–30 Hz frequency bands in channels C3 and C4 of the MA task (Figure 4A) and 8–13 Hz frequency bands in channels C3 and C4 of the MI task (Figure 4B).

Table 3 shows the channels with the average strongest ERD in the MA and MI tasks of the 14 patients. Most of the electrodes were in or around the sensorimotor areas and they presented stronger ERD than other electrodes. Besides, there was a positive correlation between the MA and MI tasks in the C4 ERD values in the 8–13 Hz (*r* = 0.534, *p* = 0.049, *n* = 14).

**TABLE 3:** Channels with average strongest ERD in the MA and MI tasks of the 14 patients.

**MA**					**MI**				
8–30 Hz	C4	CP2	CP6	P4	8–30 Hz	FP2	FP1	F4	FC2
	−10.2%	−9.3%	−8.0%	−6.4%		−5.7%	−3.1%	−2.5%	−1.9%
8–13 Hz	CP2	CP6	P4	C4	8–13 Hz	CP2	CP1	C4	Cz
	−15.6%	−15.3%	−14.2%	−13.7%		−9.8%	−8.8%	−8.3%	−7.1%
13–30 Hz	C4	CP2	CP6	FP2	13–30 Hz	FP2	FP1	F4	Fz
	−9.2%	−7.5%	−5.9%	−5.8%		−6.4%	−3.1%	−2.2%	−0.5%

### Comparison of Hemispheric Balance Between Motor Attempt and Motor Imagery

**FIGURE 5:**
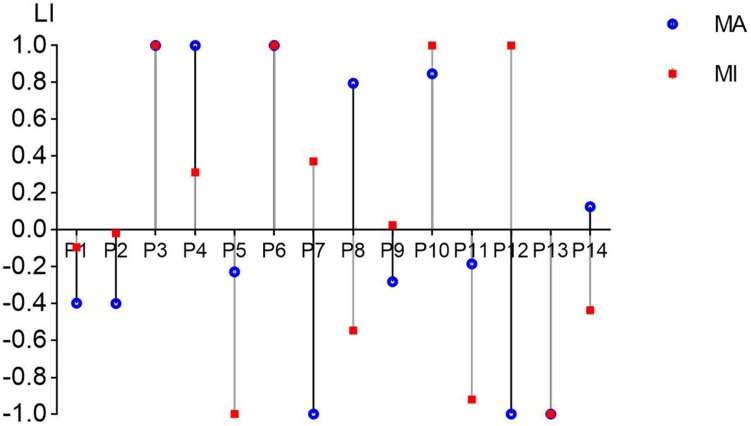
The laterality index (LI) values from C3/C4 channels in all 14 patients in the MA and MI tasks in the 8–30 Hz band. The value range of LI can be from –1 (entirely ipsilesional) to 1 (entirely contralesional). The blue round dots present the LI values of the MA task and the red square dots present the LI values of the MI task.

Figure 5 shows the LI values of all 14 patients in the MA and MI tasks in the 8–30 Hz band. Nine out of 14 patients presented the same positive/negative sign in the LI values (P3, P6, and P13 showed the same LI value between tasks) while five patients (P7, P8, P9, P12, P14) showed different positive and negative values. Eight out of 14 patients (57%) in MA and 7 out of 14 (50%) in MI showed a negative value in LI when they were performing motor tasks of wrist extension of the affected hands. There was no significant difference between the MA and MI tasks after the paired *t*-test.

### Correlations Between Brain-Computer Interface Accuracies and FMA Scores and Event-Related Desynchronization/Event-Related Synchronization Values

**FIGURE 6:**
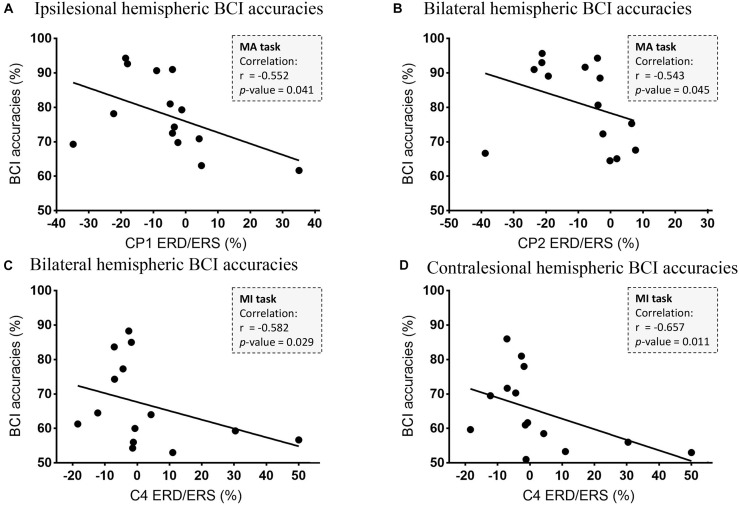
Correlations between the BCI accuracies and ERD/ERS values in the 8–30 Hz band. **(A)** The correlation between CP1 ERD and the ipsilesional hemispheric BCI accuracies (*r* = −0.552, *p* = 0.041, *n* = 14). **(B)** The correlation between CP2 ERD and the bilateral hemispheric BCI accuracies (*r* = −0.543, *p* = 0.045, *n* = 14). **(C)** The correlation between C4 ERD and the bilateral hemispheric BCI accuracies (*r* = −0.582, *p* = 0.029, *n* = 14). **(D)** The correlation between C4 ERD and the contralesional hemispheric BCI accuracies (*r* = −0.657, *p* = 0.011, *n* = 14).

Figure 6 shows the correlations between the BCI accuracies and ERD/ERS values in the 8–30 Hz band. In the MA task, there was a negative correlation between the ERD values in channel CP1 and the ipsilesional hemispheric BCI accuracies (*r* = −0.552, *p* = 0.041, *n* = 14) and a negative correlation between the ERD values in channel CP2 and the bilateral hemispheric BCI accuracies (*r* = −0.543, *p* = 0.045, *n* = 14). While in the MI task, there were negative correlations between the ERD values in channel C4 and the bilateral hemispheric BCI accuracies (*r* = −0.582, *p* = 0.029, *n* = 14) as well as the contralesional hemispheric BCI accuracies (*r* = −0.657, *p* = 0.011, *n* = 14).

For all 14 patients, there was a significant positive correlation between ipsilesional BCI accuracies and the FMA scores of the hand part in the 8–13 Hz (*r* = 0.565, *p* = 0.035, *n* = 14) in the MA task and a significant positive correlation between ipsilesional BCI accuracies and the FMA scores of the hand part in the 13–30 Hz (*r* = 0.558, *p* = 0.038, *n* = 14) in the MI task.

## Discussion

In this study, we performed two motor tasks (MA and MI) on 16 right-handed patients with hemiplegia with BCI-based experimental paradigms. We compared every subject *via* a self-control design to eliminate the potential effect of handedness. To explore the differences between the MA and MI tasks, the BCI accuracies and ERD/ERS, as well as the LI, were compared between the two tasks. It demonstrated significantly higher BCI accuracies in the MA task. Additionally, similar strength in ERD and no significant difference in the LI between the two tasks were found. The correlations between the BCI accuracies and ERD, as well as the FMA scores, were also observed.

### Difference in Brain-Computer Interface Accuracies of Motor Attempt and Motor Imagery Tasks

As it is known to all BCI researchers, MI and MA (execution) are two important experimental paradigms for motor tasks in the BCI system design. Both MI and MA have been explored in healthy subjects, and MI has also been explored in stroke or patients with hemiplegia. Thus, further exploring the BCI tasks of MI and MA is valuable in improving the clinical application of the BCI system. There were some differences between MA and MI tasks. Physically, MA was similar to motor execution and was easily accepted by stroke patients. Mentally, it was reported that MI required the active inhibition of motor neural activation, and the brain patterns during MI were less distinguishable from rest than motor execution patterns (Wolpaw et al., 2000). The patients felt that it was less natural and more difficult to perform MI. In practice, we found that the stroke patients tended to be more focused in the MA than the MI tasks and less likely to fall asleep during the motor task. Although the motor attempt was probably to induce spasticity during movement, long-term BCI studies based on the MA task as a paradigm have reported no significant increase in the spasticity of stroke patients (Biasiucci et al., 2018; Ramos-Murguialday et al., 2019). To optimize BCI application in patients with hemiplegia, we compared their differences in BCI accuracies.

The results in Figure 2 were in line with previous research (Lopez-Larraz et al., 2012; Blokland et al., 2014). Blokland et al. and Eduardo et al. both reported a significantly higher average BCI accuracy for MA tasks than MI tasks. However, their results were based on SCI patients, who had no cerebral injury. The average BCI accuracy found by Blokland et al. (2014) was 79% for MA and 70% for MI tasks. The results of the current study were similar, which was 79% for MA and 66.5% for MI tasks. The BCI accuracies of the MA task were significantly higher than those of the MI task in patients with hemiplegia. Whereas there was a difference in the BCI accuracy of the MI task in our study and Blokland’s. One explanation was that our participants were all patients with hemiplegia, whose cortical activation patterns could be different from patients with spinal cord injury. Theoretically, the BCI accuracies of SCI patients could be higher than those of patients with hemiplegia because the cortical status was relatively and functionally intact for SCI patients. The results in our study showed confidence in the BCI application for the MA task in patients with hemiplegia. Further study needs to be performed to distinguish the cortical variation between cerebral injury and SCI patients.

In recent years, the choices of EEG channels varied in BCI-related research of motor rehabilitation. The EEG signals from the bilateral (Lopez-Larraz et al., 2017), ipsilesional (Ramos-Murguialday et al., 2013; Ono et al., 2015), and contralesional (Antelis et al., 2017; Bundy et al., 2017) hemispheres were all reported to successfully control the BCI system. The different choices of EEG channels might obtain different BCI decoding effects but there was no conclusion for the best application. Our results showed no significant difference between the bilateral and unilateral hemispheres in the MA and MI tasks, but the BCI accuracies of the bilateral hemispheres were higher than those of the unilateral hemisphere in the MA task. This was consistent with Spüler et al. (2018), who explored the accuracies of the bilateral, ipsilesional, and contralesional hemispheres and found that using bi-hemispheric activity led to the best accuracies in severely paralyzed stroke patients. However, the variations in the injury location and time since the cerebral injury of our recruited patients made it difficult to find a significant difference in the BCI accuracies between the hemispheres.

### Difference in Cortical Activations of Motor Attempt and Motor Imagery Tasks

Understanding the cortical differences between MI and MA is of benefit for exploring the brain function plasticity change through BCI training since the sensorimotor rhythm (SMR)-based BCI training is based on these BCI tasks. Event-related desynchronization represents the cortical activation state and stronger ERD suggests better brain function and plasticity (Ono et al., 2015). Kraeutner et al. (2014) found that the ERD was stronger in motor execution tasks than in MI tasks in non-disabled participants. However, in our study, we only found stronger ERD with no significance in the MA task than in the MI task among the bilateral, ipsilesional, and contralesional hemispheres as well as in the C3/C4 channels (Figure 3). As MI was reported to require many of the same processes to execute (Blokland et al., 2014), the current results suggested that MA tasks might present similar cortical activity as MI tasks. Besides, the channels around the sensorimotor areas presented a stronger ERD than the average ERD in the bilateral and unilateral hemispheres during the MA task (Table 3). It was reasonable because the sensorimotor areas should be involved mostly in motor-related tasks (Li et al., 2014; Wang et al., 2016). Both fMRI and TMS showed activations in motor-related areas. Wang et al. (2016) found that there were activations among M1, bilateral premotor cortex (PMC), and supplementary motor area (SMA) in the fMRI during the motor execution and MI tasks of wrist motor control. Hummel et al. (2002) applied single-pulse TMS over the PMC (M1) and saw motor evoked potentials (MEPs) increasing when ERD occurred. The process of motor function rehabilitation was related to the motor cortex remodeling. Interestingly, as we can see in Table 3 and Figure 3, strong ERD might not only be present in sensorimotor areas (C3, C4, CP1, CP2, and CP6) but also in nearby electrodes (P4), as well as some remote electrodes (FP2), during the MA task. During the MI task, ERD was present in the sensorimotor areas but most of the strong ERD appeared in the frontal areas (F4, FP1, and FP2).

Figure 5 showed that 9 out 14 patients presented the same positive/negative sign in LI values in the MA and MI tasks. Hanakawa et al. (2008) explored different anatomical locations with fMRI in the brain and found that motor execution and MI shared neural substrates to some extent. Referring to the findings of Hanakawa, we considered that MA and MI tasks might present similar cortical excitability patterns and maintain similar activating balance between the hemispheres, although the extent of balance/LI value was not the same. As EEG is a highly non-linear process with high variability (Schomer and Da Silva, 2012), there could be some difference even in the same patient. Eight out of 14 patients (57%) in MA and 7 out of 14 (50%) in MI showed a negative value in LI in Figure 5. This finding was similar to a review (Rossini et al., 2003), although we did not apply somatosensory evoked fields detection as previous studies did. A negative/positive LI was considered to indicate a relatively stronger/lower activation in the ipsilesional hemisphere to the affected hand (Kaiser et al., 2012). Among these patients, they showed more activations in the ipsilesional hemisphere than in the contralesional hemisphere. The activations might lead to a higher BCI accuracy in ipsilesional hemispheres although there were cerebral damages on them.

### Relationship in Brain-Computer Interface Accuracies and Event-Related Desynchronization of Motor Attempt and Motor Imagery Tasks

In the previous study (Chen et al., 2021), we tried to explore the relationship between sensorimotor rhythm and upper limb motor impairment (motor dysfunction and spasticity) in MA and MI tasks. It suggests that motor dysfunction may be more correlated to ERS in the MI task and to ERD in the MA task while spasticity may be more correlated to ERD in the MA task. In this study, we focused on BCI accuracy, which is an important parameter for the BCI system. During BCI intervention, high BCI accuracies were essential to a good interaction and stronger ERD was the important foundation for cortical plasticity. Several BCI studies tried to decode motor-related signals for motor control by applying the electrodes around the sensorimotor areas (Shu et al., 2018, 2019; Spüler et al., 2018). In our study, negative correlations were found between ERD and BCI accuracies in channels CP1 and CP2 in the MA task, and between ERD and BCI accuracies in channels C4 in the MI task. These results suggested that the stronger ERD of these channels around sensorimotor areas (CP1, CP2, and C4) might lead to higher BCI accuracies. Although it is not that representative, the negative correlations between C4 as well as CP2 seem to provide evidence for those who applied BCI control by using the EEG signals from the contralesional hemispheres (Antelis et al., 2017; Bundy et al., 2017). Unlike the healthy subjects, people with brain injury may exhibit bilateral hemispheric activations due to compensation. One study (Shu et al., 2018) found that contralesional hemispheres in stroke survivors could also present activations.

These channels around the sensorimotor areas could be used to detect if the motor tasks were well performed and if the cortical excitability was modulated. Besides, it has been reported in several BCI studies that BCI accuracies were significantly associated with the improvement of upper limb motor function (Li et al., 2014) or even related to the rehabilitation efficacy of stroke patients (Bundy et al., 2017; Frolov et al., 2017). Interestingly, in the current study, there were significant positive correlations between ipsilesional BCI accuracies and the FMA scores of the hand part. However, the relationship between BCI accuracies and the improvement in motor function has not been explored in this study.

For healthy subjects, an MI classification of around 60% seems relatively low. However, it might be different in stroke survivors or people with brain injury. In a previous study (Shu et al., 2018), we found that contralesional hemispheres could also present activations, which led to a relatively low classification of around 60%. The reason might be that this activation pattern causes difficulties in distinguishing the motor tasks between the left and right hand. Up to now, there are many ways to increase BCI accuracy, among which algorithm and experimental paradigm are two of the most important methods. We think the further step for improving the current BCI accuracy may improve the experimental paradigm such as adding tactile stimulation as assistance for motor attempt tasks. One study (Shu et al., 2019) aimed at improving the SMR-based BCI accuracy by integrating motor tasks with tactile stimulation, which indicated that appropriate tactile stimulation benefited the BCI accuracy in stroke patients. It also suggested that improving the experimental paradigm can be a step to enhance BCI application. Brain-computer interface accuracies and ERD were both very important in a rehabilitative BCI system while the relationship between BCI accuracies and brain function is not totally clear. It should be further explored to get better decoding. Higher BCI accuracy is good for BCI intervention, but for stroke motor rehabilitation, the cortical response could be more important.

### Limitations of Our Study

The limitations in the study include the relatively short duration of single-trial time, the lack of long-term detection, and fMRI data. As the patients tend to be less focused than healthy subjects on the trials, the recording session was kept relatively short to minimize discomfort and to ensure that the patients were focused on the tasks. As a result, each part of one trial was set relatively short and the resting time was also short. In the beta band, the ERS might appear 300 to 500 ms after the end of the movement and last for approximately 1 s. Concurrently, in the alpha band, the power returns to the baseline after several seconds. Thus, in this study, there would not be bias in the beta band while there could have been a slight bias in the alpha band referring to the baseline (Rimbert et al., 2018, 2019b). The long-term change of MA and MI tasks has not been compared to see how BCI accuracy and ERD may change over time. Functional MRI can be added in the study to improve the spatial resolution in explaining the detailed cortical activation locations to explore the differences between MA and MI tasks.

## Conclusion

In this study, we compared the BCI accuracy and ERD/ERS, as well as LI, between MA and MI tasks in patients with hemiplegia in a self-control design. We found that the MA task achieved higher BCI accuracies than the MI task. There was no significant difference in the ERD/ERS and LI between the tasks. Cortical activation (ERD) may influence BCI accuracy, which should be carefully considered in the BCI motor rehabilitation of patients with hemiplegia.

## Data Availability Statement

The raw data supporting the conclusions of this article will be made available by the authors, without undue reservation.

## Ethics Statement

The studies involving human participants were reviewed and approved by the Ethical Committee of Huashan Hospital. The patients/participants provided their written informed consent to participate in this study.

## Author Contributions

SC and XS designed and performed the study and analyzed the data. SC organized the database and wrote the manuscript. XS, HW, LD, JF, and JJ reviewed and edited the manuscript. All authors contributed to the manuscript revision and read and approved the submitted manuscript.

## Conflict of Interest

The authors declare that the research was conducted in the absence of any commercial or financial relationships that could be construed as a potential conflict of interest.

## Publisher’s Note

All claims expressed in this article are solely those of the authors and do not necessarily represent those of their affiliated organizations, or those of the publisher, the editors and the reviewers. Any product that may be evaluated in this article, or claim that may be made by its manufacturer, is not guaranteed or endorsed by the publisher.
